# Supplementary material for: Exploring the Regulatory Effect of LPJZ-658 on Copper Deficiency Combined with Sugar-Induced MASLD in Middle-Aged Mice Based on Multi-Omics Analysis
Source: Nutrients. 2024 Jun 25;16(13):2010. doi: 10.3390/nu16132010 (PMC11243161; doi:10.3390/nu16132010)

Supplementary Material for:

Exploring the Regulatory Effect of LPJZ-658 on Copper Deficiency Combined with Sugar-Induced NAFLD in Meddle-Aged Mice Based on Multi-Omics Analysis

Chunhua Li [1], Ziqi Liu [1], Wei Wei [2], Chen Chen [1], Lichun Zhang [1], Yang Wang [3], Bo Zhou [4], Liming Liu [1], Xiao Li [4,5,*], Cuiqing Zhao [1,*]

[1] College of Animal Science and Technology, Jilin Agricultural Science and Technology University, Jilin City 132101, China.

[2] State Key Laboratory of Pathogen and Biosecurity, Beijing Institute of Microbiology and Epidemiology, Beijing 100071, China.

[3] Jilin Ginseng Academy, Changchun University of Chinese Medicine, Changchun 130117, China.

[4] Research Unit of Key Technologies for Prevention and Control of Virus Zoonoses, Changchun Veterinary Research Institute, Chinese Academy of Medical Sciences, Changchun 130122, China.

[5] Academician Workstation of Jilin Province, Changchun University of Chinese Medicine, Changchun 130117, China.

Materials and Methods

Quantitative PCR (qPCR)

Total RNA was extracted from liver tissues using RNAiso Plus (Takara Bio Inc, Kusatsu, Shiga, Japan). RNA was reverse-transcribed to cDNA using the PrimeScript TM RT reagent Kit with gDNA Eraser (Perfect Real Time) (Takara Bio Inc, Kusatsu, Shiga, Japan). mRNA expression was normalized to the 18s housekeeping gene by qPCR on an ABI StepOnePlus real-time PCR thermocycler (Thermo Fisher Scientific, Waltham, MA, USA) using TB GreenPremix Ex Taq (Tli RNaseH Plus) (Takara Bio Inc, Kusatsu, Shiga, Japan). Primer sequences are shown in Table S3.

Microbiome analysis

The collected mice cecal contents were sent for 16S rRNA sequencing at Novogene Co., Ltd. (Beijing, China). The primers 341F and 806R (Table S1) were selected to amplify the V3-V4 region of the 16S rRNA gene. Library construction and Illumina sequencing were completed by Novogene Co., Ltd. according to the manufacturer's instructions. The data were analyzed using the Novogene Magic Cloud platform (https://magic.novogene.com) .

Liver tissue lipidomic profiling by LC-MS

(1) Extraction and preparation of lipid samples

For liver lipid extraction, 50 mg of sample was homogenized in 200 μl H_2O. To each sample, 400 μl methyl tertbutyl ether (MTBE) and 80 μl methanol were added, then the samples were centrifuged (at 14,800×g for 15 min at 4 ℃) to extract lipids. The supernatant was transferred to a centrifuge tube and lyophilized using a freeze dryer. The lyophilized samples were re-dissolved in 200 μl of dichloromethane/methanol (1:1, v/v). The method for mixing quality control (QC) samples for method validation was to mix equal volumes of each sample.

(2) LC-MS analysis of lipid metabolites

Liver sample extracts were analyzed using a U3000 UPLC system coupled to a Q-Orbitrap mass spectrometer (Thermo Fisher Scientific, San Jose, CA, USA) equipped with an electrospray ionization source (ESI). The samples were analyzed using an ACQUITY UPLC BEH column (2.1×100 mm, 1.7 μm; Waters, Manchester); the column temperature was 35 ℃, and the injection volume was 2 μl. Mobile phase A consisted of 10mM $HCOONH_4$ in acetonitrile/water (6:4, v/v) and 10 mM $HCOONH_4$ in acetonitrile/2-propanol (1:9, v/v) (Phase B), with 0.3 ml/min flow rate under the following gradient program: 40 % B (0-1.5 min); 40-85 % B (1.5-10.5 min); 85 % B (10.5-14 min); 100 % B (14-14.1 min); 100 % B (14.1-15 min); 100-40 % B (15-15.2 min); 40 % B (15.2-18 min).

Mass spectrometry was operated in both the positive and negative ion modes. Profile data were acquired in the 100-1500m/z range. Tandem MS information was obtained under the DDMS2 (TOP 3) mode. The acquisition was performed at a resolution of 17,500, with ramped normalized collision energies of 30, 50, and 60. The key parameters of the ion source were set as follows: the capillary voltage was 3.5 kV in positive ion mode or -3.2 kV in negative ion mode, sheath gas flow was 50 arb, auxiliary gas flow was 10 arb, sweep gas flow was 2 arb, capillary temperature was 350 ℃. Before sample analysis, the mass spectrometer was calibrated using Pierce™ calibration solution provided by Thermo Scientific (Thermo Fisher Scientific, San Jose CA, USA). All samples were kept at 4 ℃ during analysis.

Metabolite extraction and untargeted metabolomics analysis

(1) Metabolite extraction

A volume of 50 μl of serum sample was subjected to precipitation of protein with 150 μl of methanol. The resulting mixture was then centrifuged at 12,000 rpm for 15 min at 4 ℃, and the supernatant was transferred to a centrifuge tube and lyophilized using a freeze dryer. The lyophilized samples were re-dissolved in 200 μl of methanol/water (1:1), and centrifuged at 15,000 rpm for 15 min at 4 ℃. The method for mixing quality control (QC) samples for method validation was to mix equal volumes of each sample.

(2) LC-MS analysis of serum metabolites

Serum sample extracts were analyzed using a U3000 UPLC system coupled to a Q-Orbitrap mass spectrometer (Thermo Fisher Scientific, San Jose, CA, USA) equipped with an electrospray (ESI) ionization source. The samples were analyzed using a Thermo Scientific Hyperisil Gold chromatographic column (100 mm×2.1 mm, 3 μm); the column temperature was 40 ℃, and the injection volume was 5 μl. The mobile phases are 0.1 % formic acid in water (Phase A) and 0.1 % formic acid in acetonitrile (Phase B) with 0.4 ml/min flow rate under the following gradient program: 3 % B (0-1 min); 3-70 % B (1-8 min); 70 % B (8-10 min); 70-90 % B (10-17 min); 90-100 % B (17-18 min); 100 % B (18-21 min); 100-3 % B (21-23 min); 3 % B (23-26 min). Mass spectrometry was performed in both the positive and negative ion modes. Profile data were acquired in the 100-1500m/z range. Tandem MS information was obtained under the DDMS2 (TOP 3) mode. The acquisition was performed at a resolution of 17,500, with ramped normalized collision energies of 30, 50, and 60. The key parameters of the ion source were set as follows: the capillary voltage was 3.5 kV in the positive ion mode or -3.2 kV in the negative ion mode, sheath gas flow was 50 arb, auxiliary gas flow was 15 arb, sweep gas flow was 2 arb, capillary temperature was 350 ℃. Before sample analysis, the mass spectrometer was calibrated using Pierce™ calibration solution provided by Thermo Scientific (Thermo Fisher Scientific, San Jose CA, USA). All samples were kept at 4 ℃ during analysis.

Biomarker screening

The raw LC-MS data were processed using Progenesis QI (Non-linear Dynamics, Newcastle, UK) for peak extraction, alignment, and normalization before being exported as a dataset containing the sample code, peak label, and peak intensity. Unique ions (retention time and m/z pairs) were grouped (a sum of the abundances of unique ions) using both the above molecule-related ions and isotope deconvolution to generate unique "features" (retention time and m/z pairs) representative of each compound. Data were normalized using Progenesis QI for all compounds. Annotations were assigned within Progenesis QI using accurate mass measurements, isotope distribution similarity, and manual assessment of fragmentation spectral matching (when applicable) from the LipidMaps and the Human Metabolome Database.

Pathway enrichment analysis was performed by MetaboAnalyst 5.0 service; data filtering was performed using the interquartile range (IQR), normalization by sum pairs, and Pareto scaling for data scaling, followed by multi-factor analysis, including principal component analysis (PCA) and partial least squares discriminant analysis (PLS-DA) to identify differentiated metabolites between the 2 groups.

Differences in metabolites were determined by a combination of VIP values > 1 in OPLS-DA models, p values < 0.05 in Student's t test for the comparison of peak area between groups, and fold change (FC) values of the corresponding peak area between groups $\geqslant 1.2$ or $\leqslant 0.83$.

Table S1. The information of the annotated liver lipid metabolite.

No.	Annotation	RT (min)	HMDB ID	Adducts	Detected m/z	Formula	CuDS vs. CuA	CuDS+LPJZ-658 vs. CuDS
1	20-Hydroxyeicosatetraenoic acid	1.42	HMDB0005998	[M-H]-	319.2283	C20H32O3	↑***	↓
2	PC(14:0/0:0)	1.65	HMDB0010379	[M+H]+	468.3081	C22H46NO7P	↑***	↓
3	LysoPC(20:4(5Z,8Z,11Z,14Z)/0:0)	1.67	HMDB0010395	[M+FA-H]-	588.3316	C28H50NO7P	↑***	↑
4	Glycolipids	1.67	HMDB0302365	[M+Cl]-	528.3104	C24H50N2O6P	↑***	↑
5	LysoPC(16:1(9Z)/0:0)	1.73	HMDB0010383	[M+FA-H]-	538.3159	C24H48NO7P	↑***	↓
6	LysoPC(22:5(7Z,10Z,13Z,16Z,19Z)/0:0)	1.91	HMDB0010403	[M+FA-H]-	614.3471	C30H52NO7P	↑***	↓
7	LysoPI(18:1(9Z)/0:0)	2.02	HMDB0061693	[M-H]-	597.3055	C27H51O12P	↑***	↑
8	LysoPE(0:0/22:5(4Z,7Z,10Z,13Z,16Z))	2.04	HMDB0011494	[M-H]-	526.2936	C27H46NO7P	↑***	↓
9	Retinyl ester	2.19	HMDB0003598	[M-H]-	301.2178	C20H30O2	↓***	↑
10	LysoPC(18:1(9Z)/0:0)	2.45	HMDB0002815	[M+FA-H]-	566.3469	C26H52NO7P	↑***	↓
11	LysoPC(20:2(11Z,14Z)/0:0)	2.62	HMDB0010392	[M+FA-H]-	592.3630	C28H54NO7P	↑***	↓
12	LysoPE(0:0/18:1(11Z))	2.62	HMDB0011475	[M-H]-	478.2946	C23H46NO7P	↑***	↓
13	Docosapentaenoic acid (22n-6)	3.25	HMDB0001976	[M-H]-	329.2492	C22H34O2	↑***	↑
14	Dihomo-alpha-linolenic acid	3.52	HMDB0060039	[M-H]-	305.2492	C20H34O2	↑***	↑
15	LysoPC(18:0/0:0)	3.59	HMDB0010384	[M+Cl]-	568.3629	C26H54NO7P	↑***	↓
16	LysoPC(17:0/0:0)	3.60	HMDB0012108	[M-H]-	508.3408	C25H52NO7P	↑***	↓
17	Adrenic acid	3.91	HMDB0002226	[M-H]-	331.2649	C22H36O2	↑***	↑
18	Oleic acid	4.30	HMDB0000207	[M-H]-	281.2490	C18H34O2	↑***	↓
19	Cholesterol sulfate	5.14	HMDB0000653	[M-H]-	465.3051	C27H46O4S	↑***	↑
20	PG(22:6(4Z,7Z,10Z,13Z,16Z,19Z)/22:6(4Z,7Z,10Z,13Z,16Z,19Z))	6.09	HMDB0116605	[M-H]-	865.5039	C50H75O10P	↓***	↑*
21	PG(22:6(4Z,7Z,10Z,13Z,16Z,19Z)/20:3(6,8,11)-OH(5))	6.38	HMDB0270524	[M-H2O-H]-	841.5037	C48H77O11P	↑***	↑**
22	PA(16:1(9Z)/22:4(7Z,10Z,13Z,16Z))	6.65	HMDB0114867	[M+FA-H]-	767.4882	C41H71O8P	↑***	↑

23	PC(14:0/20:4(5Z,8Z,11Z,14Z))	7.52	HMDB0007883	[M+H]+	754.5376	C42H76NO8P	↑***	↑
24	PG(18:1(11Z)/22:5(4Z,7Z,10Z,13Z,16Z))	7.88	HMDB0010627	[M-H]-	821.5341	C46H79O10P	↑***	↑
25	PI(20:4(5Z,8Z,11Z,14Z)/18:1(9Z))	8.03	HMDB0009896	[M+H]+	902.5745	C47H81O13P	↑**	↑
26	PA(14:1(9Z)/22:2(13Z,16Z))	8.07	HMDB0114805	[M+ACN+Na]+	762.5056	C39H71O8P	↑***	↑
27	PE(20:4(8Z,11Z,14Z,17Z)/16:1(9Z))	8.16	HMDB0009419	[M+FA-H]-	782.4983	C41H72NO8P	↑***	↓
28	PE(16:0/18:3(9Z,12Z,15Z))	8.24	HMDB0008930	[M+H]+	714.5064	C39H72NO8P	↓***	↑
29	PE(16:1(9Z)/18:2(9Z,12Z))	8.24	HMDB0008961	[M-H]+	712.4934	C39H72NO8P	↓***	↑
30	PC(16:0/22:6(4Z,7Z,10Z,13Z,16Z,19Z))	8.38	HMDB0007991	[M+FA-H]-	850.5613	C46H80NO8P	↓***	↓
31	PI(18:0/22:6(4Z,7Z,10Z,13Z,16Z,19Z))	8.54	HMDB0009821	[M-H]-	909.5500	C49H83O13P	↓***	↑
32	PG(16:0/20:4(5Z,8Z,11Z,14Z))	8.96	HMDB0010580	[M+ACN+H]+	812.5441	C42H75O10P	↑***	↓
33	PE(16:0/18:2(9Z,12Z))	9.03	HMDB0008928	[M+H]+	716.5219	C39H74NO8P	↓***	↑
34	PC(20:1(11Z)/22:6(4Z,7Z,10Z,13Z,16Z,19Z))	9.08	HMDB0008321	[M+H]+	860.6152	C50H86NO8P	↓***	↑
35	PA(20:1(11Z)/18:1(9Z))	9.11	HMDB0115098	[M+ACN+Na]+	792.5520	C41H77O8P	↑***	↓
36	PS(20:1(11Z)/22:2(13Z,16Z))	9.15	HMDB0112560	[M+H-2H2O]+	834.5996	C48H88NO10P	↓***	↓
37	PI(18:0/22:4(10Z,13Z,16Z,19Z))	9.29	HMDB0009817	[M-H]-	913.5823	C49H87O13P	↑***	↓
38	DG(18:1(9Z)/18:3(9Z,12Z,15Z)/0:0)[iso2]	9.32	HMDB0007221	[M+H]+	634.5399	C39H68O5	↓***	↑
39	PE(18:0/18:2(9Z,12Z))	9.72	HMDB0008994	[M+H]+	744.5527	C41H78NO8P	↓***	↓
40	DG(18:1(9Z)/20:4(5Z,8Z,11Z,14Z)/0:0)[iso2]	9.74	HMDB0007228	[M+H]+	660.5552	C41H70O5	↑***	↑
41	Cer(d18:0/16:0)	9.74	HMDB0011760	[M+FA-H]-	584.5269	C34H69NO3	↑***	↓
42	DG(16:0/20:4(5Z,8Z,11Z,14Z)/0:0)[iso2]	9.77	HMDB0007112	[M+H-H2O]+	634.5399	C39H68O5	↑***	↑
43	PC(18:0/22:4(7Z,10Z,13Z,16Z))	9.83	HMDB0008054	[M+H]+	838.6303	C48H88NO8P	↑***	↓**
44	DG(16:0/16:1(9Z)/0:0)[iso2]	9.84	HMDB0007099	[M+NH4]+	584.5243	C35H66O5	↑***	↓
45	PE(18:0/18:0)	9.86	HMDB0008991	[M+Na]+	770.5666	C41H82NO8P	↓***	↑
46	PE(18:0/20:3(5Z,8Z,11Z))	9.86	HMDB0009001	[M-H]-	768.5554	C43H80NO8P	↓***	↑
47	PE-NMe(18:0/20:4(5Z,8Z,11Z,14Z))	9.89	HMDB0113098	[M-H]-	780.5563	C44H80NO8P	↓***	↓
48	DG(18:1(9Z)/22:5(7Z,10Z,13Z,16Z,19Z)/0:0)[iso2]	9.93	HMDB0007236	[M+NH4]+	686.5713	C43H72O5	↑***	↑

49	DG(16:0/22:5(7Z,10Z,13Z,16Z,19Z)/0:0)[iso2]	9.98	HMDB0007120	[M+NH4]+	660.5548	C41H70O5	↑***	↑
50	PE-NMe(20:0/22:4(7Z,10Z,13Z,16Z))	10.01	HMDB0113302	[M+FA-H]-	882.6240	C48H88NO8P	↓***	↓
51	DG(18:1(9Z)/20:3(8Z,11Z,14Z)/0:0)[iso2]	10.02	HMDB0007227	[M+NH4]+	662.5707	C41H72O5	↑***	↑
52	PC(18:0/18:1(11Z))	10.08	HMDB0008037	[M+H]+	788.6154	C44H86NO8P	↑***	↓**
53	DG(20:1(11Z)/20:4(5Z,8Z,11Z,14Z)/0:0)[iso2]	10.17	HMDB0007402	[M+NH4]+	688.5859	C43H74O5	↑***	↑
54	Cer(d18:1/18:0)	10.18	HMDB0004950	[M+Cl]-	610.5452	C36H71NO3	↑***	↓
55	PE(18:1(11Z)/20:1(11Z))	10.18	HMDB0009032	[M-H]-	770.5714	C43H82NO8P	↓***	↑
56	PE(18:1(9Z)/20:1(11Z))	10.19	HMDB0009065	[M+H]+	772.5823	C43H82NO8P	↓***	↑
57	DG(18:0/18:2(9Z,12Z)/0:0)[iso2]	10.44	HMDB0007161	[M+H-H2O]+	638.5712	C39H72O5	↑***	↓
58	PE-NMe2(18:0/22:4(7Z,10Z,13Z,16Z))	10.45	HMDB0114005	[M-H]-	822.6052	C47H86NO8P	↑***	↓
59	DG(18:3(6Z,9Z,12Z)/24:1(15Z)/0:0)	10.65	HMDB0007297	[M-H2O-H]-	681.5798	C45H80O5	↑***	↓
60	SM(d17:1/24:1(15Z))	10.71	HMDB0011696	[M-H]-	797.6506	C46H91N2O6P	↓***	↑**
61	PE(18:0/20:1(11Z))	10.76	HMDB0008999	[M-H]-	772.5868	C43H84NO8P	↓***	↓
62	Cer(d18:1/20:0)	10.77	HMDB0004951	[M+Cl]-	638.5742	C38H75NO3	↑***	↓
63	DG(18:1(9Z)/20:1(11Z)/0:0)[iso2]	10.96	HMDB0007224	[M+NH4]+	666.6013	C41H76O5	↑***	↑
64	DG(14:1(9Z)/22:0/0:0)[iso2]	10.99	HMDB0007057	[M+H-H2O]+	605.5499	C39H74O5	↑***	↑
65	DG(18:0/18:1(9Z)/0:0)[iso2]	11.02	HMDB0007160	[M+NH4]+	640.5859	C39H74O5	↑***	↓
66	SM(d17:1/24:0)	11.06	HMDB0011695	[M+FA-H]-	845.6745	C46H93N2O6P	↓***	↓
67	Coenzyme Q9	11.44	HMDB0006707	[M+NH4]+	812.6539	C54H82O4	↓***	↑*
68	DG(24:1n9/0:0/18:2n6)	11.51	HMDB0056268	[M-H2O-H]-	697.6110	C46H84O5	↑***	↓
69	TG(16:1(9Z)/18:1(11Z)/20:5(5Z,8Z,11Z,14Z,17Z))	12.39	HMDB0048629	[M-H]-	875.7147	C57H96O6	↑***	↑
70	TG(18:1(9Z)/16:0/22:6(4Z,7Z,10Z,13Z,16Z,19Z))	12.96	HMDB0010455	[M-H]-	903.7463	C59H100O6	↑***	↑
71	TG(14:1(9Z)/18:1(11Z)/22:2(13Z,16Z))	13.09	HMDB0047944	[M+FA-H]-	927.7671	C57H102O6	↑***	↑
72	TG(18:1(9Z)/20:1(11Z)/20:1(11Z))[iso3]	14.82	HMDB0005457	[M+NH4]+	958.8785	C61H112O6	↑***	↑
73	TG(19:0/20:0/20:0)[iso3]	14.96	HMDB0064089	[M+ACN+H]+	1002.9410	C62H120O6	↑***	↑
74	TG(16:0/18:1(9Z)/20:0)[iso6]	15.13	HMDB0005381	[M+NH4]+	906.8470	C57H108O6	↑***	↓

75	PE(20:4(5Z,8Z,11Z,14Z)/16:1(9Z))	16.76	HMDB0009386	[M+FA-H]-	782.4996	C41H72NO8P	↑***	↓
76	PS(20:3(5Z,8Z,11Z)/22:2(13Z,16Z))	16.78	HMDB0112615	[M-H]-	900.4996	C48H84NO10P	↑***	↓

Arrows "↑" means up-regulated, and "↓" means down-regulated. * $p < 0.05$, ** $p < 0.01$, and *** $p < 0.001$.

Table S2. The information of the annotated serum metabolite.

No.	Annotation	RT (min)	HMDB ID	Adducts	Detected m/z	Formula	CuDS vs. CuA	CuDS+LPJZ-658 vs. CuDS
1	2-Butoxyethanol	0.83	HMDB0031327	[M+ACN+H]+	160.1321	C6H14O2	↓ *	↑ **
2	L-Valine	0.88	HMDB0000883	[M+H]+	118.0856	C5H11NO2	↓ ***	↑
3	D-Glucose	0.88	HMDB0000122	[M+Na]+	203.0512	C6H12O6	↓ ***	↓
4	2-Furanmethanol	0.90	HMDB0013742	[M+NH4]+	116.07	C5H6O2	↓ **	↑
5	Proline betaine	0.94	HMDB0004827	[M+H]+	144.1009	C7H13NO2	↓ ***	↑
6	Hypotaurine	0.94	HMDB0000965	[2M+H]+	219.046	C2H7NO2S	↓ ***	↑
7	D-Glucuronic acid	0.97	HMDB0000127	[M-H]-	193.0337	C6H10O7	↓ **	↑
8	N-Glycolylneuraminic acid	1.17	HMDB0000833	[M-H]-	324.0922	C11H19NO10	↑ *	↑ **
9	Fructose 6-phosphate	1.17	HMDB0000124	[M-H]-	259.0212	C6H13O9P	↑ ***	↓
10	Fucose 1-phosphate	1.17	HMDB0001265	[M+FA-H]-	289.0317	C6H13O8P	↓ *	↑
11	Citric acid	1.67	HMDB0000094	[M+H-2H2O]+	215.0147	C6H8O7	↓ *	↑ *
12	2-Isopropylmalic acid	1.88	HMDB0000402	[M+FA-H]-	221.0653	C7H12O5	↓ ***	↓
13	Deoxyuridine	2.07	HMDB0000012	[M-H]-	227.066	C9H12N2O5	↓ ***	↑
14	3-Deoxy-D-glycero-D-galacto-2-nonulosonic acid	2.30	HMDB0000425	[M-H]-	267.0724	C9H16O9	↓ *	↓
15	Thymidine	3.26	HMDB0000273	[M-H-	241.0818	C10H14N2O5	↓ ***	↑
16	Prunasin	3.35	HMDB0034934	[M+FA-H]-	340.1024	C14H17NO6	↓ ***	↑
17	Pantothenic acid	3.50	HMDB0000210	[M+H]-	220.1165	C9H17NO5	↓ ***	↑ ***
18	Homovanillic acid	4.05	HMDB0000118	[M-H2O-H]-	181.049	C9H10O4	↑ *	↑
19	Indolelactic acid	5.53	HMDB0000671	[M-H2O-H]-	204.0652	C11H11NO3	↑ ***	↓
20	3alpha,7alpha,12beta-Trihydroxy-5beta-cholanoic acid	6.95	HMDB0000312	[M-H]-	407.2787	C24H40O5	↓ *	↑
21	7-Ketodeoxycholic acid	7.20	HMDB0000391	[M+H-H2O]+	389.2659	C24H38O5	↓ *	↑
22	Leukotriene B4	7.79	HMDB0001085	[M-H2O-H]-	335.2216	C20H32O4	↑ *	↑

23	Oleic acid	7.82	HMDB0000207	[M-H]-	281.2474	C18H34O2	↑***	↓
24	Sphingosine 1-phosphate	8.59	HMDB0000277	[M-H]-	378.24	C18H38NO5P	↓**	↑*
25	12S-HHT	9.21	HMDB0012535	[M+H-H2O]+	263.1987	C17H28O3	↓**	↑**
26	5,6-Epoxy-8,11,14-eicosatrienoic acid	11.03	HMDB0002190	[M-H]-	319.2265	C20H32O3	↑***	↑
27	15(S)-Hydroxyeicosatrienoic acid	11.55	HMDB0005045	[M+H-H2O]+	305.2453	C20H34O3	↑***	↑
28	Octanoylcarnitine	11.97	HMDB0000791	[M+NH4]+	305.2451	C15H29NO4	↑***	↑
29	Eicosapentaenoic acid	13.03	HMDB0001999	[M-H]-	301.216	C20H30O2	↓***	↑
30	Docosahexaenoic acid	13.61	HMDB0002183	[M+H-H2O]+	329.2451	C22H32O2	↓***	↓
31	Arachidonic acid	13.93	HMDB0001043	[M-H]-	303.2318	C20H32O2	↑***	↓
32	Linoleic acid	14.25	HMDB0000673	[M+H]+	263.2351	C18H32O2	↓*	↑
33	Citrulline	19.51	HMDB0000904	[M+NH4]+	193.1298	C6H13N3O3	↓**	↑*

Arrows " ↑ " means up-regulated, and " ↓ " means down-regulated. * $p < 0.05$, ** $p < 0.01$, and *** $p < 0.001$.

Table S3. Primer Sequences for q-PCR analysis

Gene	Source	Sequences (Forward/Reverse 5'-3')	
Sphk1	Mouse	AGGTGGTGAATGGGCTAATG	TGCTCGTACCCAGCATAGTG
Sphk2	Mouse	GTACTCATGTTGGGCATCTT	CATACTCCACTAACTCCCCA
Cers2	Mouse	TCATCCCTTCTCAGTATTGGT	ATCCTTTCGCTTGACATCAG
Cers4	Mouse	ACCCTGAATTTGTCCCTGTA	CTTGAAGTCCTTGCGTTTG
Cers6	Mouse	TGTGCCATAGCCCTCAAC	CTCCGAACATCCCAGTCC
Sgms1	Mouse	GGTCGTCCATGAACGAGTA	TGAAATAGCCAGAGTCCTACAA
Smpd1	Mouse	GCCTGCAAAGTCTTATTCAC	CACCACATCGTCCTCAAAG
Smpd2	Mouse	GCCCAGTTCATCCACCAC	CCTCAGTCTCAACGAAAGC
Smpd3	Mouse	TCATGGACGTGGCCTATC	ACCTGCACCTTGAGAAACAG
Smpd4	Mouse	GGAATCTCCGATGCCTACA	ATCATTGGACCACTTGGGT
Sptlc1	Mouse	TACGAGGCTCCAGCATACC	TCAGAACGCTCCTGCAACT
Sptlc2	Mouse	CCATGCGTCACTGGTTCTA	GTCCGAGGCTGACCATAAA
18s	Mouse	CTAACCCGTTGAACCCCATT	CCATCCAATCGGTAGTAGCG
341F		CCTAYGGGRBGCASCAG	
806R		GGACTACNNGGGTATCTAAT	

Figure S1. Representative base peak chromatograms of Liver sample extracts ((A), ESI+ and (B), ESI−).

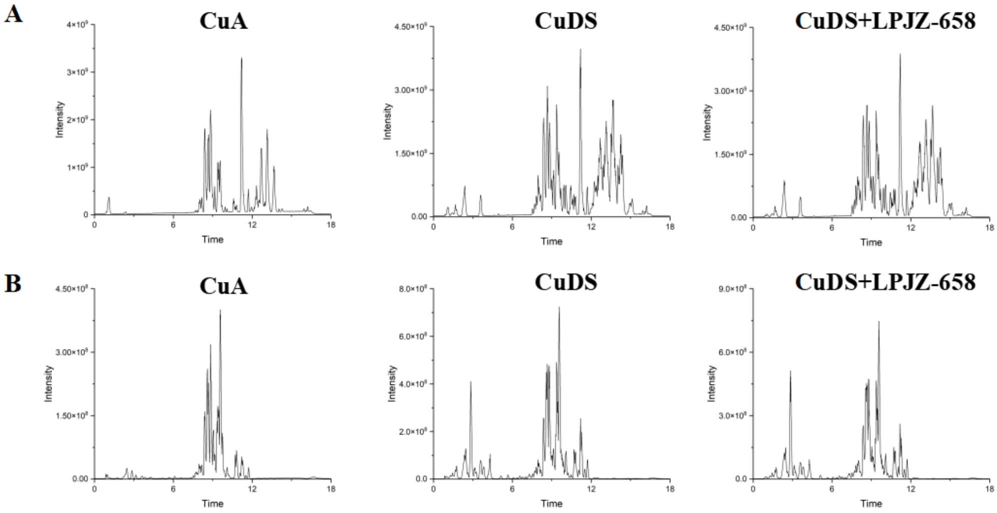

Figure S2. Representative base peak chromatograms of serum sample extracts ((A), ESI+ and (B), ESI−).

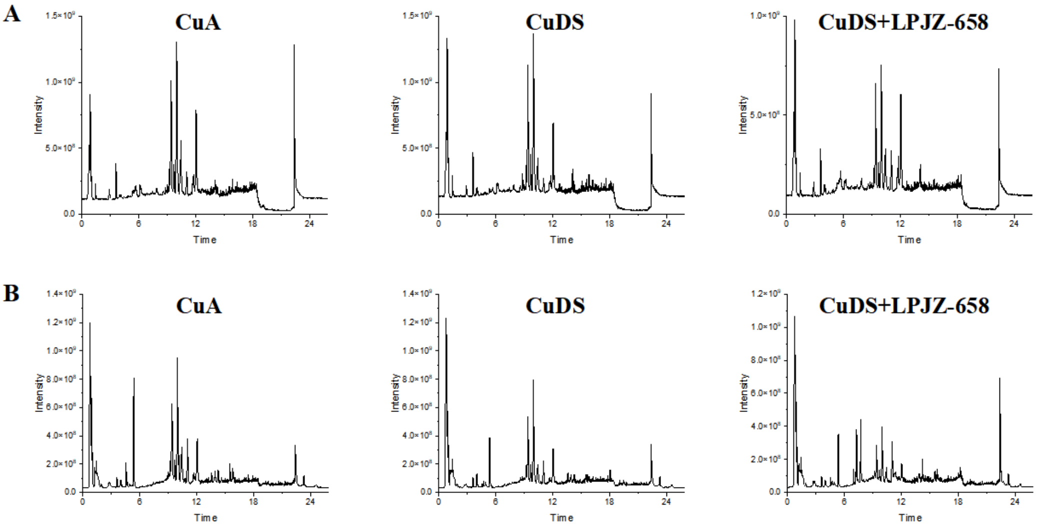

Figure S3. Relative abundance of significantly different bacterial genera in cecum samples detected by LEfSe. (A) Erysipelotrichales; (B) Erysipelotrichaceae; (C) Faecalibaculum; (D) Rodentium; (E) Dubosiella; (F) Proteobacteria; (G) Gammaproteobacteria; (H) Alphaproteobacteria; (I) Pseudomonadales; (J) unidentified_Bacteria; (K) Desulfovibrionia; (L) Desulfovibrionale; (M) Desulfovibrionaceae. Solid and dashed lines indicate mean and median, respectively.

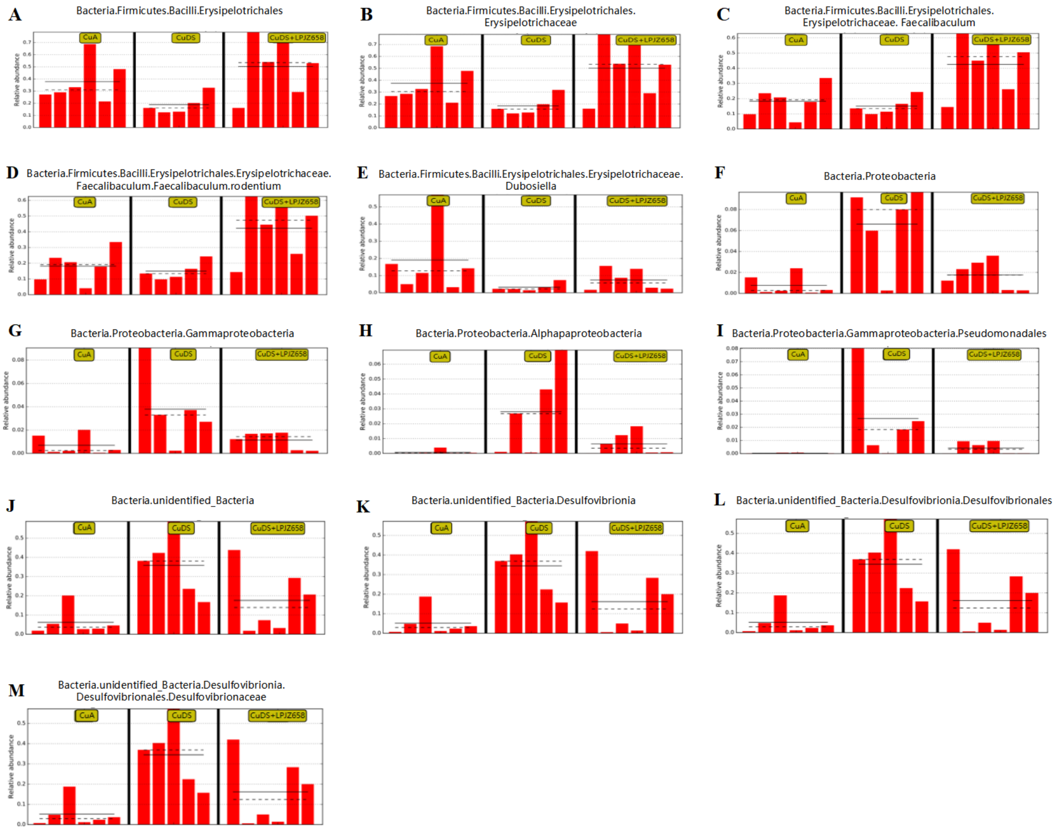

Supplement: Supplementary file 1 [file nutrients-16-02010-s001.zip › nutrients-3020293-supplementary.pdf]
